# Diet-Quality and Socio-Demographic Factors Associated with Non-Nutritive Sweetener Use in the Australian Population

**DOI:** 10.3390/nu10070833

**Published:** 2018-06-27

**Authors:** Amanda Grech, Chi On Kam, Luke Gemming, Anna Rangan

**Affiliations:** Nutrition and Dietetics Discipline, School of Life and Environmental Sciences at the Charles Perkins Centre, The University of Sydney, Sydney 2006, Australia; amanda.grech@sydney.edu.au (A.G.); ckam4454@uni.sydney.edu.au (C.O.K.); luke.gemming@sydney.edu.au (L.G.)

**Keywords:** intense sweeteners, artificial sweeteners, sweetening agents, sweetened beverages, diet quality, nutrition, diabetes, obesity

## Abstract

Non-nutritive sweeteners (NNS) are used in the food supply to replace sugar and/or to reduce dietary energy intake. The aim of this research was to assess the consumption prevalence and food sources of NNS in the Australian population. Food group and nutrient intakes were assessed to compare diet quality of NNS consumers and non-consumers. Secondary analysis of the Australian National Nutrition and Physical Activity Survey, 2011/12 was conducted (*n* = 12,435) after identifying all NNS products consumed in the population. The proportion of participants that reported intake of NNS per day was 18.2% for adults (19+ years), and 8.5% for children (2–18 years), with the most common food sources being carbonated soft drinks, tabletop sweeteners, and yoghurt. Characteristics associated with NNS consumption in adults included being female, higher body mass index (BMI), self-reported diabetes status, and being on a weight-loss diet. For adults, NNS consumers had lower free sugar intake but energy intake did not differ from non-consumers. However, for children, no differences in free sugar or energy intake were observed between consumers and non-consumers. While these results support the use of NNS in reducing sugar intake, these data suggest compensatory increases in energy intake may occur.

## 1. Introduction

Non-nutritive sweeteners (NNS) are energy-free or very low energy alternatives to sugar and other nutritive sweeteners, and are often marketed as ‘diet’, ‘sugar-free’, ‘no sugar’, or ‘reduced sugar’. In Australia, sweeteners are classified as food additives and the declaration of any sweeteners used in the ingredient list is mandatory [1]. Common NNS include saccharin, cyclamate, aspartame, acesulphame K, sucralose, alitame, neotame, and stevia [2]. NNS offer much higher intensity of sweetness per unit weight than sucrose and can be found in a wide range of food and beverage products such as yoghurt, jam, chewing gum, cordials, and soft drinks. They are also sold as tabletop sweeteners, which can be added to tea, coffee, or used in baking recipes to replace sugar.

When NNS were first introduced, they were believed to be beneficial to reduce sugar and energy intake for weight management [3,4,5], but study findings on their efficacy are inconsistent [6,7,8,9]. Adverse health effects of NNS consumption have been suggested, primarily based on animal studies [10,11,12] and observational studies [13,14] which have raised public awareness and negative perception towards NNS, potentially influencing their consumption.

Sylvetsky et al. examined the consumption prevalence of NNS in the United States (US) and observed an increasing trend in both adults and children between 1999 and 2008 [15]. NNS consumption prevalence in the US population was found to be higher in females, the obese, non-Hispanic whites, and those with higher incomes [16]. In Australia and New Zealand, a survey of apparent high NNS consumers was undertaken in 2003 and determined that the current level of NNS consumption was below the acceptable daily intake (ADI) [17]. However, the analyses did not investigate the overall and population-specific consumption prevalence of NNS [17]. No other studies have evaluated NNS consumption in Australia for the past 15 years. Therefore, the aim of this study was to: (1) investigate the consumption prevalence of NNS in Australia and the current consumption pattern of NNS across different population subgroups; (2) identify the major dietary sources of NNS; and (3) determine differences in diet quality between NNS consumers and non-consumers.

## 2. Materials and Methods

### 2.1. Data Collection

This study used dietary and physical activity data from the National Nutrition and Physical Activity Survey (NNPAS), 2011–12, a cross-sectional study involving 12,153 participants aged 2 years or above in Australia. The dietary component involved two 24-h recalls of all foods and beverages consumed on the day before the interview, adopting the Automated Multiple-Pass Method developed by the United States Department of Agriculture. The first recall was face-to-face (*n* = 12,153), and the second recall (*n* = 7735) was conducted over the phone at least 8 days after the first recall. Children aged 2–4 years were interviewed by proxy, and recalls for 5–11 year-old children were conducted with assistance from their parents or guardians. The detailed descriptions of the survey, conducted by the Australian Bureau of Statistics (ABS), including data collecting and processing procedures are available from the Users’ Guide [18].

### 2.2. Coding

Foods or beverages containing NNS or reported as an ingredient (for example intense sweetening agents used to sweeten foods or beverages) were identified using the Food Details File of AUSNUT 2011-13 [19,20]. This file provides descriptive information of 5740 food and beverage items reported in the NNPAS 2011-12. The search terms diet, intense sweeten(ed), artificial sweeten(ed), non-nutritive sweeten(ed), reduced sugar, sweetener, stevia, sucralose, acesulfame K, aspartame, saccharin, neotame, cyclamate, alitame, low energy, no sugar, and sugar-free were used to identify food items containing NNS. The description of some food types such as meal replacements, protein drinks, protein powder, and other sports supplements was unclear regarding the presence of NNS. For these food types, the ingredient lists of the most common brands were examined to determine the presence or absence of NNS. All NNS-containing items were coded and merged with the NNPAS 2011-12 dataset to identify participants who reported NNS consumption.

### 2.3. Statistical Analysis

The prevalence of NNS consumption per day was determined by the proportion of participants who reported consuming at least one NNS-containing food or beverage in the first 24-h recall. Characteristics associated with NNS consumption were determined for children (aged 2–18 years) and adults (aged 19 years and over) using chi-squared tests. Demographic and health-related characteristics analyzed included gender, age, country of birth, socioeconomic status (SES), body mass index (BMI), physical activity level, and energy reporting status for children and adults, and education level, diabetes status, smoking status, and weight loss diet adoption for adults only. Socio-Economic Indexes for Areas (SEIFA) quintiles were used to indicate SES (quintile 1 as low, quintiles 2–4 medium, and quintile 5 high) [18]. Energy reporting status was determined using the ratio of energy intake (EI) to basal metabolic rate (BMR). Participants with EI:BMR less than 0.87 or more than 2.67 were classified as low energy reporters and high energy reporters, respectively.

The major dietary sources of NNS were identified by comparing the proportions of the population consuming different types of NNS-containing food and beverage items. Participants who reported consumption of NNS-containing items in at least one of the two recalls were classified as consumers and the rest were classified as non-consumers. The potential effects of NNS consumption on diet quality were investigated by comparing the mean intake of energy, 10 nutrients (carbohydrates, total sugar, free sugar, total fat, saturated fat, monounsaturated fatty acids, polyunsaturated fatty acids, protein, sodium, and dietary fibre) and 6 food groups (grains, meat and alternatives, dairy and alternatives, fruits, vegetables, and discretionary foods) of consumers and non-consumers using independent *t*-tests. Data were weighted to be representative of the national population aged 2 years and over. IBM SPSS Statistics for Windows (Version 25.0) was employed for all statistical analyses. A *p*-value was set at <0.001 because of the large number of analyses undertaken.

## 3. Results

A total of 122 out of 5740 food items in the AUSNUT2013 database were identified as NNS-containing foods. The prevalence of NNS consumption across different population subgroups is shown in Table 1 (children) and Table 2 (adults). Overall, 8.5% of children and 18.2% of adults reported NNS consumption in the first 24-h recall.

### 3.1. Population Subgroups Comparisons

For children, the consumption prevalence increased with age (Table 1). Children born in English-speaking countries other than Australia were higher consumers than those born in Australia and other countries.

For adults, consumption of NNS was higher in females than males, and for those born in Australia and other English-speaking countries (Table 2). The prevalence was higher with increasing BMI, for participants with self-reported diabetes, and for those on a weight-loss diet. In addition, low energy reporters were more likely to consume NNS than plausible reporters and high energy reporters.

### 3.2. Major Dietary Sources of NNS

**Figure 1:**
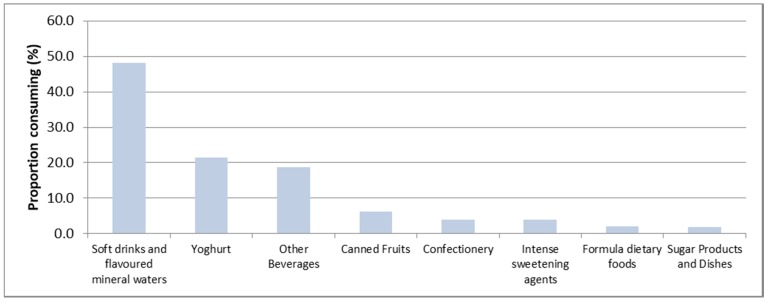
Dietary sources of non-nutritive sweeteners (NNS) among children NNS consumers.

The most popular dietary sources of NNS for children were carbonated soft drinks and flavored mineral waters (48.2% of NNS consumers), followed by yoghurt (21.5%), and other beverages (18.7%) such as cordials and electrolyte drinks (Figure 1).

**Figure 2:**
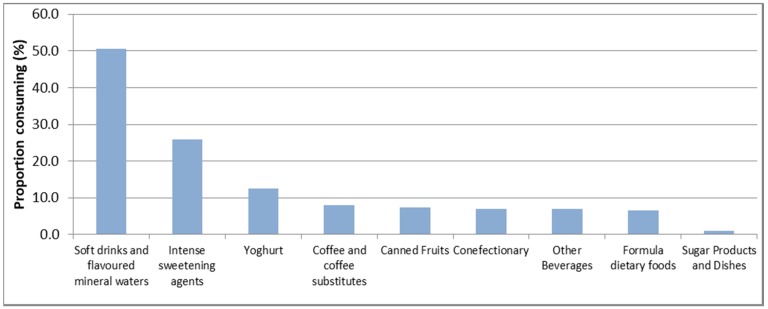
Dietary sources of NNS among adult NNS consumers.

For adults, the top dietary sources of NNS were carbonated soft drinks and flavoured mineral waters (50.6% of NNS consumers), followed by intense sweetening agents (25.9%), and yoghurt (12.5%) (Figure 2).

### 3.3. Food and Nutrient Intakes

For children, male consumers reported higher intakes of protein, meat and alternatives, and dairy and alternatives (Table 3). No significant differences in either nutrient or food group intakes were observed between female consumers and non-consumers.

For adults, the free sugar intake was significantly lower and the intakes of protein and sodium higher for male consumers of NNS (Table 4). Similarly, significantly lower intakes of free sugar and significantly higher protein intake were observed among adult female consumers in addition to lower carbohydrate intake. In terms of food groups, significantly higher intake of dairy products and alternatives was observed in both male and female adult consumers.

## 4. Discussion

The results of the present study showed that the consumption of NNS was about twice as prevalent in Australian adults (18.2%) than in children (8.5%). In children, NNS consumption prevalence increased with age. This increasing trend continued over the entire lifespan, reaching a prevalence of 1 in 5 adults consuming NNS. Other characteristics associated with NNS consumption included being female, higher BMI, self-reported diabetes status, being on a weight-loss diet. Carbonated soft drinks, yoghurt, and intense sweetening agents were the main dietary sources of NNS in the Australian diet. NNS consumers reported similar energy intakes compared with non-consumers but adult consumers had lower intakes of free sugars than non-consumers.

Similar trends across age, gender, and BMI status have been reported in the US by Sylvetsky et al. [16]. No association was observed in the present study with socioeconomic status or education, unlike in the US [21]. Consumption of NNS is seen as an effective way to limit sugar and energy intake, which is reflected in higher consumption patterns in females, with older age, higher BMI, and for those on weight-loss diets in adults [22]. Concern with body image and diet quality may be underlying factors for the higher consumption of NNS among adolescents and (female) adults [23,24,25]. The higher consumption of NNS among people with diabetes was expected, in order to manage blood glucose levels. As many as 96% of patients with diabetes surveyed in Mexico used NNS [26].

Our results indicated that beverages were the main dietary contributors of NNS for children and adults. The most frequently consumed NNS-containing beverages were carbonated soft drinks and flavoured mineral water with half of the NNS consumers reporting these on one of the two recall days. These findings were consistent with the results from a study in the US [23] and a study published by the Food Standards Australia New Zealand (FSANZ) in 2004 [17], that both identified carbonated soft drinks as the top NNS contributors. Tabletop sweetening agents were also a major contributor to NNS consumption among adults but not children. This difference may be explained by the higher intakes of coffee and tea by adults, with tabletop intense sweetening agents replacing tabletop sugar. In terms of foods, the most commonly consumed NNS source was yoghurt for both children and adults, and this may have contributed to the higher intakes of protein from dairy products in NNS consumers.

Although there was no difference in energy intake for adults, there was an observed reduction in intake of free sugars in NNS consumers compared with non-consumers. This is consistent with experimental evidence that suggests that NNS consumers may consume more energy in subsequent meals to compensate for the reduced energy intake [8,27]. However, this tendency was not evident among children with no significant differences for intakes of total or free sugars between NNS consumers and non-consumers. At present it is unclear whether NNS can assist in improving glycaemia or weight management and conversely their use has been implicated in the development of obesity and metabolic disorders [6,14]. However, the data for these studies are primarily observational and likely confounded by other factors [6,8,14]. Two recent reviews found positive associations between body weight and consumption of NNS but conversely found that randomized controlled trials (RCT) that replaced sugar with NNS led to decreased body weight [5,9]. Observational studies are subject to reverse causation and may explain the conflicting results between observational and RCT [22]. Interpreting the evidence base is further complicated as much of the research in NNS is funded by industry, which has introduced bias that cannot be explained by other sources [28]. Additional long-term, well designed human studies are needed to determine the effects of NNS on health and wellbeing [5].

Other differences in diet quality were noted between consumers and non-consumers of NNS, such as higher intakes of protein, meat, and dairy and alternatives, and sodium, and lower intakes of carbohydrates among some consumer sub-groups. Other population-based studies that have examined the diet quality of NNS consumers found that diet quality of consumers was higher than that of non-consumers [25,29]. However, another study that only included obese patients found the opposite [30]. The results of the present study demonstrate that NNS consumers also misreported energy intakes, were on a weight-loss diet, or reported a current diagnosis of diabetes mellitus, and these groups may report higher diet quality [31,32]. The population group chosen for comparison may explain the conflicting results.

The prevalence of daily NNS consumption in Australia is likely attributed to both public health campaigns (and national dietary guidelines) emphasizing the importance of limiting free sugar intake [33] and the frequent introduction of new NNS-containing food and beverages to the market during this time [17]. Additionally, a recent increase in the use of Stevia across a broad range of products in the Australian food supply will likely further change consumption patterns of NNS in the future. As such, it is expected that NNS consumption will continue to rise in the future. This highlights the need to continue investigating the potential benefits and/or health consequences of NNS consumption.

## 5. Limitations

As the dietary data in the present study was collected using self-reported 24-h recalls, the consumption prevalence is subject to bias [34]. Recall bias may be present because of respondents’ memory lapses or intentionally avoid reporting common NNS sources of other foods assessed in this analysis [32]. In addition, the daily consumption prevalence of NNS was determined based on one 24-h recall and therefore may not reflect the usual intake of the participants due to day-to-day variations [35]. Future studies should consider conducting multiple recalls or assess NNS with food frequency questionnaires (FFQ) to better capture the consumption prevalence and frequency of intake of the population.

## 6. Conclusions

A considerable proportion of the Australian adult population consume NNS, in particular by those that may be using NNS for weight-loss and control of glycaemia in diabetes mellitus. In adults, NNS consumers achieved lower free sugar intake, relative to the non-consuming population. However, there was no significant reduction in total energy intake, implying compensatory increases in energy intake from other food sources. While these results add to the evidence that NNS may be useful in decreasing free sugar intake, at present, the benefits of consuming NNS on body-weight and metabolic health is unclear. Future research to evaluate the effectiveness of NNS and guide its application in different population groups is required.

## Figures and Tables

**Table 1:** Prevalence of non-nutritive sweetener consumption per day of Australian children aged 2–18 years.

Characteristics	Prevalence (%)	*p*-Value
**All**	8.5	-
**Age (years)**		
2–3 (*n* = 464)	4.8	<0.0001 *
4–8 (*n* = 789)	4.9	
9–13 (*n* = 787)	9.7	
14–18 (*n* = 772)	11.4	
**Gender**		
Male (*n* = 1420)	8.0	0.3105
Female (*n* = 1392)	9.0	
**Socioeconomic status**		
Low (*n* = 478)	6.0	0.0773
Medium (*n* = 1626)	9.3	
High (*n* = 708)	8.4	
**Country of birth**		
Australia (*n* = 2530)	7.9	<0.0001 *
Other English-speaking countries (*n* = 123)	20.8	
Other (*n* = 159)	8.8	
**Body mass index (BMI) status**		
Underweight (*n* = 123)	9.9	0.0231
Normal (*n* = 1595)	7.5	
Overweight (*n* = 427)	11.3	
Obese (*n* = 158)	12.3	
**Physical activity**		
Meets recommendations (*n* = 1195)	7.9	0.6567
Does not meet recommendations (*n* = 1617)	7.4	
**Energy reporting status**		
Low (*n* = 261)	14.8	0.0005
Plausible (*n* = 1956)	8.1	
High (*n* = 101)	4.8	

* Significant difference (*p* < 0.001) analyzed using *X*^2^ test.

**Table 2:** Prevalence of non-nutritive sweetener consumption per day of Australian adults aged 19 years and over.

Characteristics	Prevalence (%)	*p*-Value
**All**	18.2	-
**Age (years)**		
19–30 (*n* = 1592)	16.0	0.0178
31–50 (*n* = 3565)	18.5	
51–70 (*n* = 2906)	19.4	
71 and over (*n* = 1278)	19.0	
**Gender**		
Male (*n* = 4282)	16.8	<0.0003 *
Female (*n* = 5059)	19.7	
**Socioeconomic status**		
Low (*n* = 1760)	19.4	0.3347
Medium (*n* = 5547)	18.1	
High (*n* = 2134)	17.6	
**Education**		
High school or less (*n* = 3636)	18.3	0.0854
Vocational college (*n* = 2193)	18.0	
University (*n* = 3369)	18.6	
**Country of birth**		
Australia (*n* = 6627)	20.2	<0.0001 *
Other English-speaking countries (*n* = 1152)	17.6	
Other (*n* = 1562)	11.9	
**BMI status**		
Underweight (*n* = 116)	7.2	<0.0001 *
Normal (*n* = 2678)	12.6	
Overweight (*n* = 2883)	18.9	
Obese (*n* = 2196)	25.7	
**Physical activity**		
Meets recommendations (*n* = 4593)	19.1	0.0423
Does not meet recommendations (*n* = 4678)	17.4	
**Self-reported diabetes**		
Yes (*n* = 640)	38.4	<0.0001 *
No (*n* = 8701)	17.0	
**Weight-loss diet**		
Yes (*n* = 615)	35.1	<0.0001 *
No (*n* = 8726)	17.0	
**Smoking**		
Yes (*n* = 1769)	17.3	0.2846
No (*n* = 7572)	18.4	
**Energy reporting status**		
Low (*n* = 1587)	24.0	<0.0001 *
Plausible (*n* = 6200)	17.3	
High (*n* = 137)	10.3	

* Significant difference (*p* < 0.001) analyzed using *X*^2^ test.

**Table 3:** Dietary intake of children (aged 2–18) non-nutritive sweeteners (NNS) consumers and non-consumers.

	Male			Female		
	C	NC	*p*-value	C	NC	*p*-value
Dietary component						
Energy (kJ)	8300	8578	0.3117	7404	7212	0.3896
Nutrients						
Carbohydrates (%E)	48.1	50.1	0.0045	50.6	49.9	0.2880
Total Sugars (%E)	21.2	22.7	0.0276	24.4	23.0	0.0387
Free Sugar (%E)	11.3	13.2	0.0048	13.4	12.7	0.3064
Total Fat (%E)	30.9	31.0	0.9586	30.7	31.2	0.4039
Saturated Fat (%E)	13.5	13.7	0.6564	13.8	13.6	0.5630
MUFA (%E)	11.5	11.3	0.5186	10.9	11.4	0.0970
PUFA (%E)	4.1	4.1	0.8603	4.0	4.2	0.1458
Protein (%E)	18.1	16.1	<0.0001 *	15.7	16.1	0.3869
Sodium (mg)	2479	2489	0.8964	2002	2038	0.5795
Fibre (%E)	2.0	2.0	0.3230	2.0	2.1	0.1348
Food Groups						
Discretionary Foods (%E)	32.7	37.0	0.0132	36.9	36.1	0.6092
Grains and Cereals (serves)	4.8	4.8	0.8514	3.9	3.9	0.6923
Meat & Alternatives (serves)	1.6	1.2	<0.0001 *	0.9	1.0	0.3615
Dairy & alternatives (serves)	2.1	1.6	0.0001 *	1.5	1.4	0.3213
Fruit (serves)	1.5	1.8	0.1438	1.8	1.6	0.2588
Vegetables (serves)	1.7	1.9	0.2168	1.3	1.6	0.0195

* Significant difference (*p* < 0.001) analyzed using independent *t*-tests. C; consumers; NC; non-consumers; %E; percentage energy; MUFA; monounsaturated fats; PUFA; poly-unsaturated fats.

**Table 4:** Dietary intake of adults (aged 19 years and over) NNS consumers and non-consumers.

	Male			Female		
	C	NC	*p*-value	C	NC	*p*-value
Dietary component						
Energy (kJ)	9665	9863	0.1573	7238	7408	0.0684
Nutrients						
Carbohydrates (%E)	42.8	43.4	0.1154	42.3	43.8	<0.0001 *
Total Sugars (%E)	18.1	18.7	0.0789	19.2	19.9	0.0209
Free Sugar (%E)	9.2	10.7	<0.0001 *	9.2	10.3	<0.0001 *
Total Fat (%E)	30.5	30.3	0.5978	31.1	31.3	0.4329
Saturated Fat (%E)	11.9	12.0	0.4937	11.9	12.2	0.1099
MUFA (%E)	11.8	11.6	0.2112	11.9	11.9	0.8910
PUFA (%E)	4.7	4.6	0.1968	5.0	5.0	0.5831
Protein (%E)	19.5	17.9	<0.0001 *	19.9	18.1	<0.0001 *
Sodium (mg)	2838	2677	0.0005 *	2112	2062	0.0979
Fibre (%E)	2.2	2.1	0.2967	2.3	2.4	0.0613
Food Groups						
Discretionary Foods (%E)	33.7	34.4	0.307	30.4	30.6	0.8011
Grains and Cereals (serves)	5.0	5.2	0.0912	3.7	3.9	0.0011
Meat and Alternatives (serves)	2.2	2.1	0.3305	1.7	1.6	0.0139
Dairy and alternatives (serves)	1.7	1.4	<0.0001 *	1.5	1.3	<0.0001 *
Fruit (serves)	1.7	1.5	0.0496	1.4	1.5	0.0474
Vegetables (serves)	2.9	3.2	0.0013	2.8	3.0	0.0034

* Significant difference (*p* < 0.001) analyzed using independent *t*-tests. C; consumers; NC; non-consumers; %E; percentage energy; MUFA; monounsaturated fats; PUFA; poly-unsaturated fats.
